# Causal effect of shifting from precarious to standard employment on all-cause mortality in Sweden: an emulation of a target trial

**DOI:** 10.1136/jech-2023-220734

**Published:** 2023-08-23

**Authors:** Nuria Matilla-Santander, Anthony A Matthews, Virginia Gunn, Carles Muntaner, Bertina Kreshpaj, David H Wegman, Néstor Sánchez-Martínez, Julio C Hernando-Rodriguez, Maria Albin, Rebeka Balogh, Letitia Davis, Theo Bodin

**Affiliations:** 1 Unit of Occupational Medicine, Karolinska Institute, Stockholm, Sweden; 2 Unit of Epidemiology, Institute of Environmental Medicine, Karolinska Institutet, Stockholm, Sweden; 3 MAP Centre for Urban Health Solutions, Li Ka Shing Knowledge Institute, Unity Health Toronto, Toronto, Ontario, Canada; 4 School of Nursing, Cape Breton University, Sydney, New South Wales, Canada; 5 Bllomberg Faculty of Nursing, Division of Social and Behavioural Sciences, University of Toronto, Toronto, Ontario, Canada; 6 Department of Public Health, University of Copenhagen, Copenhagen, Denmark; 7 Public Health, University of Massachusetts Lowell, Lowell, Massachusetts, USA; 8 Public Health, Universitat Internacional de Catalunya, Barcelona, Spain; 9 Centre for Occupational and Environmental Medicine, Stockholm Region, Stockholm, Sweden; 10 Interface Demography, Vrije Universiteit Brussel, Brussels, Belgium; 11 Institute for Employment Research, University of Warwick, Coventry, UK; 12 Researcher, Cambridge, Massachusetts, USA

**Keywords:** OCCUPATIONAL HEALTH, MORTALITY, EPIDEMIOLOGY, LONGITUDINAL STUDIES

## Abstract

**Background:**

We aimed at estimating the causal effect of switching from precarious to standard employment on the 6-year and 12-year risk of all-cause mortality among workers aged 20-55 years in Sweden.

**Methods:**

We emulated a series of 12 target trials starting every year between 2005 and 2016 using Swedish register data (n=251 273). We classified precariously employed individuals using a multidimensional approach at baseline as (1) remaining in precarious employment (PE) (73.8%) and (2) shifting to standard employment (26.2%). All-cause mortality was measured from 2006 to 2017. We pooled data for all 12 emulated trials and used covariate-adjusted pooled logistic regression to estimate intention-to-treat and per-protocol effects via risk ratios (RRs) and standardised risk curves (the parametric g-formula).

**Results:**

Shifting from precarious to standard employment decreases the 12-year risk of death by 20% on the relative scale (RR: 0.82, 95% CI: 0.73; 0.93), regardless of what happens after the initial shift. However, we estimated a 12-year risk reduction of 30% on the relative scale for workers shifting from precarious to standard employment and staying within this employment category for the full 12 years (RR: 0.71, 95% CI: 0.54; 0.95).

**Conclusions:**

This study finds that shifting from low to higher-quality employment conditions (ie, stable employment, sufficient income levels and high coverage by collective agreements) decreases the risk of death. Remaining in PE increases the risk of premature mortality. Our results emphasise the necessity of ensuring decent work for the entire working population to accomplish the 2030 Agenda for Sustainable Development.

**WHAT IS ALREADY KNOWN ON THIS TOPIC**

- Evidence suggests that single components of precarious employment (mostly temporary employment and low income) are associated with an increased risk of all-cause mortality.
- Most of the evidence on precarious employment and mortality comes from ecological and cross-sectional studies, which use one-point time exposure measurements, being susceptible to recognised biases and reverse causation.

**WHAT THIS STUDY ADDS**

- This is the first study to examine the causal effect of shifting from precarious to standard employment on all-cause mortality. The statistical methods used allow us to reduce biases present in the previous literature.
- This study estimated a 12-year mortality risk reduction of 30% on the relative scale for workers shifting from precarious to standard employment and staying within this employment category for the full 12 years.

**HOW THIS STUDY MIGHT AFFECT RESEARCH, PRACTICE OR POLICY**

- Our results showing decreased risk of all-cause mortality for workers moving from precarious to non-precarious employment support the need for multilevel interventions aimed at reducing employment insecurity and income inadequacy and increasing workers’ rights and protection.

## Introduction

Precarious employment (PE) is a well-known social determinant of health that contributes to the creation of health inequalities.^1^ Yet, mortality associated with PE has not been explored sufficiently and high-quality longitudinal studies are lacking. PE is low-quality employment characterised by employment insecurity, income inadequacy, and lack of rights and protection.^2^ For example, a worker in PE would be that one who is employed through an agency (employment insecurity), has low-income levels (income inadequacy) and cannot exercise his/her working rights (lack of rights and protection). While PE is at the lower end of the employment quality continuum, the standard employment relationship is generally considered good-quality employment at the upper part of this continuum. Standard employment is commonly described as full-time and permanent employment that is based on a hierarchical employer/employee relationship that includes explicit benefits and rights.^1^

Previous studies that focused on specific components of PE such as temporary employment or income have found an increased risk of mortality compared with permanently employed workers. A Finnish study found lower death risk for workers transitioning from temporary to permanent employment.^3^ This is in line with a study conducted in France that found an increased risk of mortality among temporarily employed males compared with permanent workers.^4^ Another study conducted in Belgium described several non-standard employment arrangements (temporary agency, seasonal, fixed-term employment) to be associated with a higher mortality risk relative to permanent employment, especially among men.^5^ Moreover, a study conducted in the USA described associations between income volatility and all-cause mortality.^6^ All these studies show evidence that certain characteristics of PE increase the risk of mortality. The potential explanations behind this increased risk are that workers with characteristics of PE suffer from economic insecurity, material deprivation and chronic stress and poorer working conditions.^7^

All these studies draw attention to the detrimental health effects of PE by using one-dimensional exposures (single components of PE). But, since PE is a multidimensional phenomenon characterised by multiple low-quality employment characteristics, it requires multidimensional measurements that cover all these characteristics. For instance, a recent meta-analysis concluded that being exposed to single components of PE for 12 months and the risk of all-cause mortality was imprecisely estimated.^8^

Furthermore, previous evidence exploring the all-cause mortality risk associated with PE is mostly based on ecological and cross-sectional studies that are susceptible to recognised biases (ecological fallacy, length-biased sampling, recall bias, common method bias, etc) and reverse causation. Also, most of the studies use one-point time exposure measurements and do not consider possible changes in the employment status of the workers.

Therefore, the aim of this study was to estimate the causal effect of shifting from precarious to standard employment at ages 20-55 years on the 6-year and 12-year risk of all-cause mortality among precariously employed workers in Sweden.

## Methods

### Study design and data collection

We emulated a series of target trials starting every year between 2005 and 2016 using the Swedish Work, Illness, and Labour-market Participation (SWIP) cohort (see table 1).^9^ The SWIP cohort is the result of the linkage of multiple registers and includes all registered individuals in Sweden, aged 16–65 years in 2005 and onwards until December 2017 (approximately 5.7 million individuals). The SWIP cohort also includes information on previous health conditions, socioeconomic characteristics and employment of the included individuals. For this study, the follow-up of individuals started at baseline and stopped on the day of death or the end of the study (December 2017), whichever happened first.

**Table 1:** Specification and emulation of the target trial of precariously employed workers who either remain in precarious employment or shift to standard employment and mortality risk using Swedish register data

Protocol component	Target trial specification (randomised pragmatic trial)	Target trial emulation (using observational data)
Eligibility criteria	To have been at least the year immediately prior in precarious employment (defined by a precarious employment score of <−3) Age 20–55 Not studying Having a yearly income >100 SEK Being unemployed 0 or <3 months in a year Not being self-employed (solo or non-solo) The baseline is defined as the first year in which all eligibility criteria are met (2005–2016)	Same as for the target trial specification
Treatment strategies	Shifting to standard employment (precarious employment score >−1) at baseline and remaining in it during follow-up Remaining in precarious employment (precarious employment score <−3) over follow-up	Same as for the target trial
Treatment assignment	Study participants are randomly assigned to a strategy at baseline, and they will be aware of the assigned treatment strategy	We classified study participants according to the strategy that their data were compatible with at baseline and attempted to emulate randomisation by adjusting for baseline confounders (we assume that groups are exchangeable at baseline conditional on baseline covariates)
Outcomes	All-cause mortality	Same as for the target trial specification
Follow-up	Starts at baseline and ends at the year of death, loss to follow-up (emigration), end of follow-up (December 2017), whichever happens first	Same as for the target trial
Causal contrasts	Intention-to-treat effect (the effect of assignment to standard employment vs precarious employment at baseline, regardless of future employment during follow-up); here we will measure a point intervention Per-protocol effect (the comparative effect of following the ‘treatment strategies’ specified in the study protocol); here we will measure a sustained intervention	Observational analogue of intention-to-treat and per-protocol effect
Statistical analysis	Intention-to-treat analysis: risk ratios and standardised risk curves by means of pooled logistic regression model with time-fixed ‘treatment’ variable. With this model, we compare the risk between those who shift to standard employment and those who remain in precarious employment at baseline (regardless of future employment during follow-up). Per-protocol analysis: participants are censored when they deviate from their assigned treatment strategy (this also includes switching to other employment states, such as self-employment) and use inverse-probability weights to adjust for pre-baseline and post-baseline prognostic factors associated with adherence.	Intention-to-treat analysis: same as for the target trial but also including baseline variables in the pooled logistic regression model. Per-protocol analysis: same as for the target trial. Individuals will be ‘artificially’ censored when they deviate from their assigned strategy (inverse probability weights for artificial censoring).

SEK, Swedish krona.

Included registers were:

- Longitudinal integrated database for health insurance and labour market studies register (we used data from 2003 to 2016): information on socioeconomic variables, employment/work-related variables.
- Death register (we used data from 2005 to 2017): information on the date of death.
- National Patient Register for specialist inpatient and outpatient care (we used data from 2005 to 2016): including the date of diagnosis and cause of diagnosis (the 10th revision of the International Statistical Classification of Diseases and Related Health Problems; ICD-10 codes).

Individuals were included in the study if at baseline: (1) they had been in at least the year immediately prior to PE (defined by a PE score of <−3), (2) they were aged 20–55 years old (young ages are included because of higher share of workers in PE and different causes of death than older ages), (3) they were not studying (to avoid misclassification of students into PE), (4) they had been unemployed 0 or less than 3 months in a year (to capture the effect of PE on mortality instead of unemployment), (5) they were not self-employed (also to avoid misclassification of certain types of self-employed into PE), (6) the PE score was not between −3 and –1 (individuals in this range are excluded to make clear the contrast between PE and standard employment), (7) they had the full information for classifying employment as precarious or not. The baseline is defined as the first year in which all the eligibility criteria are met for all 12 target trials. For example, for the baseline year of 2005, study participants must have been at least in 2004 in PE and had to fulfil the rest of the inclusion criteria for 2005.

### Variables

#### Exposure variable (treatment strategies)

We classified individuals in PE into two strategies according to baseline information:

1. Shifting to standard employment at baseline and remaining in it during follow-up.
2. Remaining in PE over follow-up.

For measuring PE and standard employment, we used the Swedish Register-based Operationalization of Precarious Employment (SWE-ROPE).^10^ SWE-ROPE covers the three dimensions (employment insecurity, income inadequacy and lack of rights and protection) of PE as identified by Kreshpaj *et al*.^2^ The components are:

- Contractual employment insecurity score: ranges from −1 to 0, as follows: −1 (employed through an agency) and 0 (directly employed).
- Temporariness score: ranges from −2 to 0, as follows: −2 (unstable employment) and 0 (stable employment). Measured as a change in the employer in the past 2 years or the current year.
- Multiple job holding score: ranges from −2 to 0, as follows: −2 (multiple jobs and multiple sectors), −1 (multiple jobs), 0 (no multiple jobs). Measured as having one or more employers in the current year and the economic sector of these employers.
- Income-level score: ranges from −2 to 2, as follows: −2 (income <60% of the median), −1 (income 60–80% of the median), 0 (income 81–120% of the median), 1 (income 121–200% of the median), 2 (income >200% of the median). Measured based on work-related income, work-related social insurance benefits (parental benefits, sickness benefits and related sources) and unemployment benefits.
- Probability of unionisation score: based on the probability of coverage under collective bargaining agreements; ranges from −2 to 0, as follows: −2 (<70%), −1 (from 70–90%), 0 (>90%). For the years 2014–2016, the probability of 2013 was used because this was the last year that such data were available.

The sum of the score resulted in a summative score ranging from −9 to 2 (PE score). A detailed description and discussion regarding the operationalisation can be found in a previous paper.^11^ We considered an individual to be in PE in a year when the total score was <−3, and to be in standard employment when their total score was >−1.

#### Outcome variable

All-cause mortality retrieved from the Swedish Cause of Death Register.

#### Other variables

We obtained the minimal sufficient set of variables for adjustment by drawing the causal assumptions using a Directed Acyclic Graph (online supplemental figure 2). Confounders were sex, age, level of education, country of birth, days in unemployment in the year, family composition and health disorders (measured as any health disorder diagnosed in specialised inpatient and outpatient care, which does not include minor health conditions neither primary care visits and excluding the following categories ‘O: pregnancy, childbirth and the puerperium’, ‘P: certain conditions originating in the perinatal period’, ‘Q: congenital malformations, deformations and chromosomal abnormalities’). The adjustment of health disorders at baseline is done to rule out reverse causation and previous exposures to PE.

10.1136/jech-2023-220734.supp1 Supplementary data

### Statistical analysis

We emulated a series of 12 target trials (starting each year between 2005 and 2016), such that everyone may participate in multiple target trials.^12–16^ This approach was taken as an individual may meet the eligibility criteria several times over follow-up. A positive side product increased statistical efficiency.

For the treatment assignment, we attempted to emulate randomisation by adjusting for baseline confounders (we assumed that groups are exchangeable at baseline conditional on the baseline covariates). We pooled the data for all 12 emulated target trials and estimated intention-to-treat and per-protocol effects via risk ratios (RRs) and standardised risk curves by means of logistic regression.

We calculated two causal contrasts: the intention-to-treat effect and the per-protocol effect (table 1). The intention-to-treat effect measures the effect of ‘assignment’ to shift to standard employment compared with remaining in PE at baseline. This effect measures a point intervention, meaning that it measures the baseline effect of shifting to standard employment on mortality regardless of future employment changes over the follow-up. Alternatively, the per-protocol effect measures the effect of following in the assigned treatment of baseline (standard employment or PE) over the follow-up. This effect measures a sustained intervention.

For the intention-to-treat effect, we fitted a pooled logistic regression model containing an indicator of observed treatment initiation, the baseline covariates and a flexible function of years since assignment (linear and quadratic terms). For estimating the risk curves and differences in the 6-year and 12-year risk, we standardised for baseline covariate distribution. We calculated counterfactual risk curves and used them to estimate the RR, cumulative incidence, and risk difference at 6 and 12 years.

For the per-protocol effect, we censored individuals when they deviated from their treatment group (ie, when they did not adhere to their baseline exposure). We then calculated the inverse probability of adherence weights using pooled logistic regression models. With these models, we calculated the predicted probabilities of adhering to the treatment strategy assigned at baseline. The aim for calculating these weights was to attempt to ‘remove’ any association between adherence and treatment strategy, conditional on the baseline and time-varying predictors of adherence. We estimated the weights separately for each exposure group (remaining in PE and shifting to standard employment) because the reasons for non-adherence may differ between the groups. We calculated stabilised truncated weights at the 99th percentile. Next, we calculated risk curves standardised for baseline covariates (as in the intention-to-treat effect) and weighted for baseline and time-varying predictors of adherence.

We used bootstrapping with 500 samples to calculate 95% CIs for incidence and incidence differences estimates. For RR estimates, we used robust variances to calculate 95% CIs.

Further, we repeated the previous analyses stratifying the sample by sex (women and men) and age groups at baseline (20–39 and 40–55 years old) to study the potential modifier effects of these variables on the effect estimates.

The code for estimating the intention-to-treat and per-protocol effects is available at: https://github.com/nuriamatillasantander/The-causal-effect-of-switching-from-precarious-to-standard-employment-on-all-cause-mortality.

## Results

This study includes 251 273 workers (online supplemental figure 1 and online supplemental table 1) in PE who either remained in PE at baseline (73.8%) or shifted to standard employment at baseline (26.2%) (table 2). The sociodemographic characteristics of both groups at baseline are similar. The proportion of those who had been unemployed at baseline was higher in the group who remained in PE at baseline (14%), while having a higher level of education was higher in those who shifted to standard employment at baseline (37%).

**Table 2:** Baseline characteristics of study participants, Sweden (n=251 273), 2005–2016

Characteristics	N (%)	Remain in precarious employment (n=185 480)	Shift to standard employment (n=65 794)
Participants	251 273	73.8%	26.2%
Age (mean, SD)		34.9 (10.1)	34.4 (8.6)
Sex (% women)	148 978 (59.3)	59.2	59.4
Level of education (% higher education >3 years)	50 737 (20.2)	14.2	37.1
Country of birth (% Sweden)	218 550 (86.3)	86.3	88.8
Family composition (% single with children)	28 560 (11.4)	11.9	9.8
Yearly unemployment (1–90 days)	28 942 (11.5%)	13.9	4.6
Health disorders*	85 915 (34.2%)	34.4	33.7

Baseline ranges from 2005 to 2016. Each individual may contribute to more than one trial.

*Health disorders correspond to any health disorder diagnosed in specialised inpatient or outpatient care (not including primary care visits), excluding the chapters ‘O: pregnancy, childbirth and the puerperium’, ‘P: certain conditions originating in the perinatal period’, ‘Q: congenital malformations, deformations and chromosomal abnormalities’.

**Figure 1:**
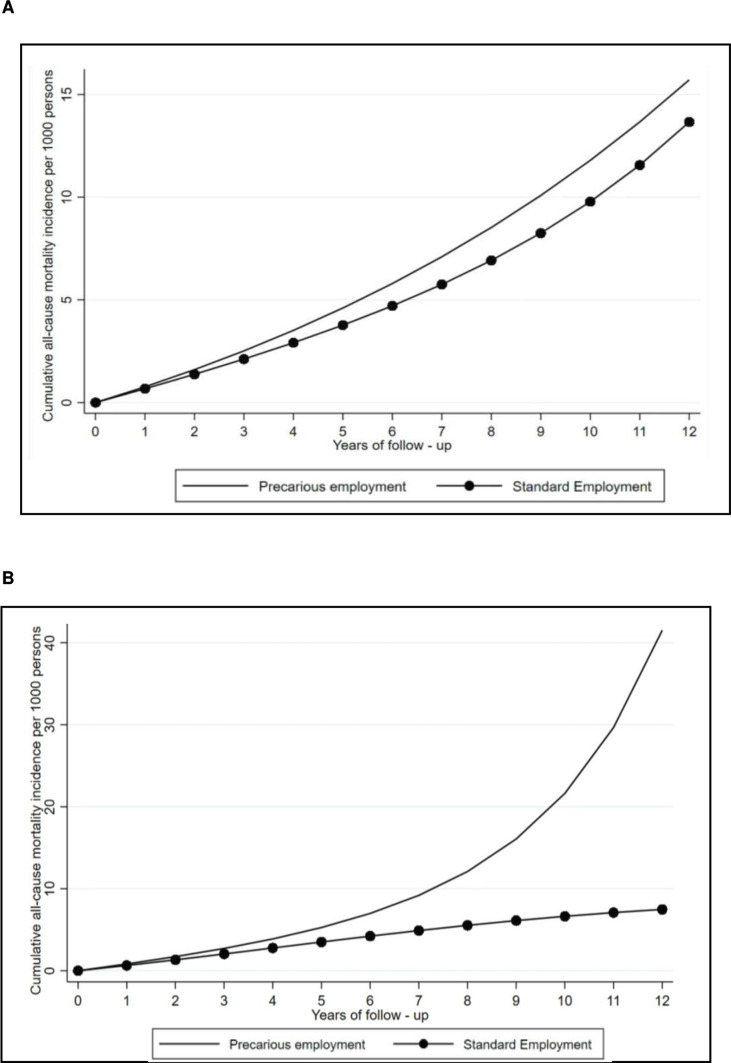
Standardised risk curves comparing shifting to standard employment versus remaining in precarious employment, 2005–2017. (A) Observational analogue to an intention-to-treat analysis. Risk curves standardised for baseline covariate distribution. Note: risk curve standardised for baseline covariate distribution (age, level of education, sex, health diagnoses, family composition, unemployment spells). (B) Observational analogue to per-protocol analysis. Risk curves standardised for baseline covariate distribution and weighted for time-varying confounders. Note: risk curve standardised for baseline covariate distribution (age, level of education, sex, health diagnoses, family composition, unemployment spells) and weighted for time-varying confounding.

Figure 1 shows the cumulative incidence of all-cause mortality per 1000 persons over follow-up in those who remained in PE and those who shifted to standard employment at baseline (A), and the risk of individuals who remained in the same group over the follow-up (B). The intention-to-treat effect (A) shows a higher risk of mortality among those who remained in PE at baseline (being the 6-year incidence of 5.79 deaths per 1000 persons compared with 4.70 in those who shift to standard employment). Similarly, the per-protocol effect (B) shows a higher risk of mortality among those who remained in PE over the follow-up, or in other words, did not shift to standard employment at baseline and remained in PE the whole follow-up (being the 6-year incidence of 6.87 deaths per 1000 persons compared with 4.05 in those who shifted to standard employment).

Along these lines, shifting from precarious to standard employment at baseline (intention-to-treat effect) decreases the risk of death on the relative scale by 20% (RR: 0.82, 95% CI: 0.73; 0.93) (table 3). The 6-year and 12-year mortality incidence difference is 1 (−1.09, 95% CI: −1.88; −0.29) and 2 (−2.05, 95% CI: −3.57; −0.53) less deaths per 1000 persons. When adhering to standard employment over the entire follow-up (per-protocol effect), the reduction in risk on the relative scale is 30% (RR: 0.71, 95% CI: 0.54; 0.95), being the 6-year incidence difference of 3 (−2.83, 95% CI: −5.03; −0.63) fewer deaths per 1000 persons, respectively (table 3).

**Table 3:** Estimated 6-year and 12-year standardised incidence differences and risk ratios for all-cause mortality comparing shifting to standard employment with no shifting among precariously employed workers, 2005–2017 (n=251 273)

		Remaining in precarious employment	Shifting to standard employment
Deaths, n (%)		1244 (0.67)	316 (0.48)
Intention-to-treat effect*	6-year mortality incidence per 1000 persons (95% CI)	5.79 (5.56; 6.03)	4.70 (3.94; 5.47)
	6-year mortality incidence difference (95% CI)	Ref	−1.09 (−1.88; −0.29)
	12-year mortality incidence per 1000 persons (95% CI)	15.71 (14.97; 16.45)	13.66 (12.19; 15.13)
	12-year incidence difference (95% CI)	Ref	−2.05 (−3.57; −0.53)
	RR (95% CI)	Ref	0.82 (0.73; 0.93)
Per-protocol effect†	6-year mortality incidence per 1000 persons (95% CI)	6.87 (5.36; 8.38)	4.05 (2.09; 5.98)
	6-year mortality incidence difference (95% CI)	Ref	−2.83 (−5.03; −0.63)
	12-year mortality incidence per 1000 persons (95% CI)	41.5 (−20.16; 103.21)	7.48 (−33.11; 48.07)
	12-year mortality incidence difference (95% CI)	Ref	−34.04 (−92.14; 24.05)
	RR (95% CI)	Ref	0.71 (0.54; 0.95)

RR: adjusted for age, level of education, sex, health diagnoses, family composition, unemployment spells at baseline. Mortality incidence and differences were standardised to the joint distribution of the baseline covariates for the intention-to-treat effect. For the per-protocol effect, they were also weighted for time-varying confounders.

*Comparing shifting to standard employment versus no shifting at baseline.

†Comparing shifitng to standard employment at baseline and continuation over follow-up.

RR, risk ratio.

The adherence to the exposure (treatment assignment) at baseline, which is remaining in the same exposure that individuals had at baseline, was 39% (online supplemental table 2). The adherence was much lower among individuals who remained in the PE group (29%) compared with those who shifted to standard employment (67%) at baseline. Therefore, the 12-year risk estimates in the per-protocol analysis are to be interpreted with caution.

Regarding the stratified results by sex and age, it is observed that the decrease of the risk of death on the relative scale is quite similar among men and women (table 4), as well as in the different age groups (online supplemental table 3). However, it is important to highlight that men have a higher 6-year and 12-year all-cause mortality incidence and incidence differences compared with women (table 4).

**Table 4:** Estimated 6-year and 12-year standardised incidence differences and risk ratios for all-cause mortality for men and women comparing shifting to standard employment with no shifting among precariously employed workers, 2005–2017 (n=251 273)

		Men		Women	
		**Remaining in precarious employment**	**Shifting to standard employment**	**Remaining in precarious employment**	**Shifting to standard employment**
Deaths, n (%)		715 (0.95)	173 (0.65)	529 (0.48)	143 (0.37)
Intention-to-treat effect*	6-year mortality incidence per 1000 persons (95% CI)	8.35 (7.85; 8.84)	6.78 (5.48; 8.08)	4.04 (3.79; 4.30)	3.32 (2.62; 4.01)
	6-year mortality incidence difference (95% CI)	Ref	−1.57 (−2.81; −0.32)	Ref	−0.73 (−1.38; −0.08)
	12-year mortality incidence per 1000 persons (95% CI)	21.32 (19.9; 22.7)	17.75 (15.1; 20.4)	11.90 (11.01; 12.79)	10.95 (9.14; 12.77)
	12-year incidence difference (95% CI)	Ref	−3.57 (−6.34; −0.79)	Ref	−0.95 (−2.76; 0.86)
	RR (95% CI)	Ref	0.81 (0.69; 0.96)	Ref	0.85 (0.70; 1.03)
Per-protocol effect†	6-year mortality incidence per 1000 persons (95% CI)	10.13 (7.07; 13.19)	6.27 (3.64; 8.91)	4.86 (3.03; 6.70)	3.18 (1.53; 4.83)
	6-year mortality incidence difference (95% CI)	Ref	−3.86 (−7.58; −0.13)	Ref	−1.68 (−3.60; 0.24)
	RR (95% CI)	Ref	0.69 (0.48; 1.02)	Ref	0.76 (0.50; 1.14)

RR: adjusted for age, level of education, health diagnoses, family composition, unemployment spells at baseline. Mortality incidence and differences were standardised to the joint distribution of the baseline covariates for the intention-to-treat effect. For the per-protocol effect, they were also weighted for time-varying confounders.

*Comparing initiation to standard employment versus no initiation at baseline.

†Comparing initiation to standard employment at baseline and continuation over follow-up. The 12-year mortality incidence and incidence difference are not shown for the per-protocol effect, due to very low adherence and therefore low cases, which produced unreliable effects.

RR, risk ratio.

## Discussion

This study estimates that the effect of shifting from precarious to standard employment decreases the 12-year risk of death by 20% on the relative scale, regardless of what happens after the initial shift (ie, one could move back to PE), being the 6-year and 12-year mortality incidence difference of shifting from precarious to standard employment of 1 and 2 deaths per 1000 people, respectively. However, we estimated a 12-year risk reduction of 30% on the relative scale for workers shifting from precarious to standard employment and staying within this employment category for the full 12 years, with a 6-year mortality incidence difference of 3 deaths per 1000 people.

The absolute risk of death may seem to be low in the intention-to-treat effect (1 and 2 deaths per 1000 people) and in the per-protocol effect (3 deaths per 1000 people). But considering that the population all-cause mortality is rather low at the baseline age of 20–55 years old (0.7 deaths per 1000 people in 2005 in Sweden), the mortality difference observed in shifting from precarious to standard employment is quite large. This means that remaining in PE increases the risk of premature mortality, generating avoidable premature deaths. Based on our results, these deaths could be avoided if workers had been exposed to higher-quality employment conditions (ie, being directly employed, having stable employment, holding one job, having medium or high-income levels and a high probability of coverage by collective agreements).

Moreover, we observed similar relative risks of all-cause mortality among men and women, although the differences in the mortality incidence were higher in men compared with women, being of four less deaths among men who shift to standard employment at baseline and stay within this employment category for the full 6 years compared with one less death among women. In previous studies conducted in Sweden, it has also been observed a larger effect of remaining in PE on cardiovascular disorders among men compared with women.^17^ Possible explanation for this is men have poorer lifestyle factors compared with women, and a possible interaction between PE and occupational risk factors among male-dominated occupations.

Our results are in line with previous literature that examined the mortality risk associated with specific components of PE. PE may increase the risk of death through several pathways. One of the pathways would be economic insecurity and material deprivation. Precariously employed workers tend to have unstable employment positions along with low-income and volatile-income levels, and, as a result, they are more likely to suffer from economic insecurity and, therefore, material deprivation.^18^ Both economic insecurity and material deprivation have been linked to an increased risk of premature mortality.^19–21^

Another pathway through which PE could increase the risk of mortality is the combination of irregular working hours and poor work–life balance.^22–24^ PE is linked more commonly with irregular working hours, such as shift, night shift work and long working hours (since the earnings from a standard work week would tend to be insufficient). Shift work^25^ and long working hours^24^ have been associated with increased risk of cardiovascular death^24^ through the disruption of circadian rhythms and increased risk of cardiovascular disease, through trigger-related pathways linking long working hours to increased coagulation, atrial arrhythmia, heavy alcohol consumption and disruption of social/private life.

PE may also increase the risk of death through exposure to chronic stress. Previous cross-sectional studies have described a higher prevalence of self-reported stress in Europe and the production of adrenal hormones (a biomarker of stress) among precariously employed workers in Spain^26^ (both studies using multidimensional measurements of PE). This may be explained by the exposure to occupational stress, as well as stress related to material deprivation, economic uncertainty and job insecurity. Chronic stress has been also associated with an increased risk of mortality from cardiovascular disease^27^ and all-cause mortality.^28^

Another hypothesised pathway through which PE affects health is through exposure to workplace hazards (chemicals, noise, heat or cold, vibrations and psychosocial risks). Precariously employed workers tend to be at higher risks in the workplace because they are over-represented in occupations with numerous workplace hazards. They also have fewer rights and protection in the workplace, which in turn, decreases their access to personal protective equipment and safety training, which has been linked with all-cause mortality.^29^

Finally, PE has been linked with an increased risk of mental and occupational injuries, so these associated morbidities could increase the risk of all-cause mortality.^30–32^

Our results show that PE is a highly relevant determinant of health. The next research steps are to explore the specific causes of mortality and to conduct mediation analyses to understand which are the main mechanisms that explain the increased risk of mortality. Given the increasing trends of PE, our results emphasise the important role of decent employment conditions for the health of the working population going forward. Our results support the necessity to achieve decent work for all the working population, as flagged in the 2030 Agenda for Sustainable Development.

### Limitations and strengths

This study has some limitations. Exposure measurement is yearly based and therefore changes over the year cannot be well captured. Since we excluded self-employed, our results are only applicable to salaried workers. The adherence was much lower among individuals who remained in the PE group (29%). This is not surprising, since a sizeable share of individuals in PE tends to shift to higher employment quality positions over time. But the low adherence is the reason why the risks later in follow-up in the per-protocol analyses are widely different, obtaining imprecise estimates for the 12-year mortality risks. Therefore, the 12-year mortality risks estimated for the per-protocol analysis should be interpreted with caution, and our interpretation of the per-protocol analysis is based on the 6-year mortality risk estimates. By censoring individuals who change exposure over time, it is possible to rule out the effect of changing exposures during the follow-up, and therefore overcome a huge limitation of previous studies.

On the other hand, this study uses high-quality register data. By using g-methods, we can control for time-varying confounding and for treatment-confounder feedback and, as a result, we can minimise bias for adjusting for confounders that are related to previous exposure (eg, the time-varying confounders level of education or family composition).^16^ Further, we can also minimise reverse causation (ill health predicts worse employment quality and vice versa). There is the possibility of unobserved effect of social disadvantage in the group of workers in PE that may explain the mortality risk among this group. However, we are adjusting by disadvantage factors that account for that, such as changes in family composition (changes in marital status, number of children), level of education, days in unemployment in the year and health disorders, as well as country of birth, age and sex that are predictors of other social disadvantage variables. Therefore, we are confident that we are adjusting by social disadvantage factors that may explain the mortality risk among workers in PE. Moreover, accumulated social disadvantage such as material and social deprivation in precariously employed workers is one of the mediators through which they have overall higher mortality, and therefore we are not adjusting for that.

Further, in this study, we are also accounting for history of PE (1 year before entering the study) and other covariates that are also caused by previous exposure to PE (eg, health disorders, days in unemployment).

## Conclusion

We estimated a 12-year risk reduction of 30% on the relative scale for workers shifting from precarious to standard employment and staying within this employment category for the full 12 years. Given the increasing trends of PE, our results emphasise the important role of decent employment conditions for the health of the working population going forward.

## Data Availability

Data may be obtained from a third party and are not publicly available. The data underlying the results of this study are available from Statistics Sweden and the Swedish National Board of Health and Welfare and were used by the current study under license, after ethical review.
