# Supplementary material for: Causal effect of shifting from precarious to standard employment on all-cause mortality in Sweden: an emulation of a target trial
Source: J Epidemiol Community Health. 2023 Aug 23;77(11):736–43. doi: 10.1136/jech-2023-220734 (PMC10579471; doi:10.1136/jech-2023-220734)
Supplement: Supplementary data [file jech-2023-220734supp001.pdf]

The causal effect of shifting from precarious to standard employment on all-cause mortality in Sweden: an emulation of a target trial.

Figure S1. Flow-chart of the study population (page 2)

Table S1. Sample size at baseline for all the series of target trials included (page 3)

Figure S2. Directed Acyclic Graph (DAG) for baseline confounders. (page 4)

Table S2. Adherence to the baseline exposure across time and by exposure group. (page 5)

Table S3. Estimated six and 12-year standardized incidence differences and risk ratios for all-cause mortality for age groups 20 to 39 years old, and 40 to 55 years old, comparing initiation to standard employment with no initiation among precariously employed workers, 2005-2017 (n= 251273). (page 6)

Figure S1. Flow-chart of the study population.

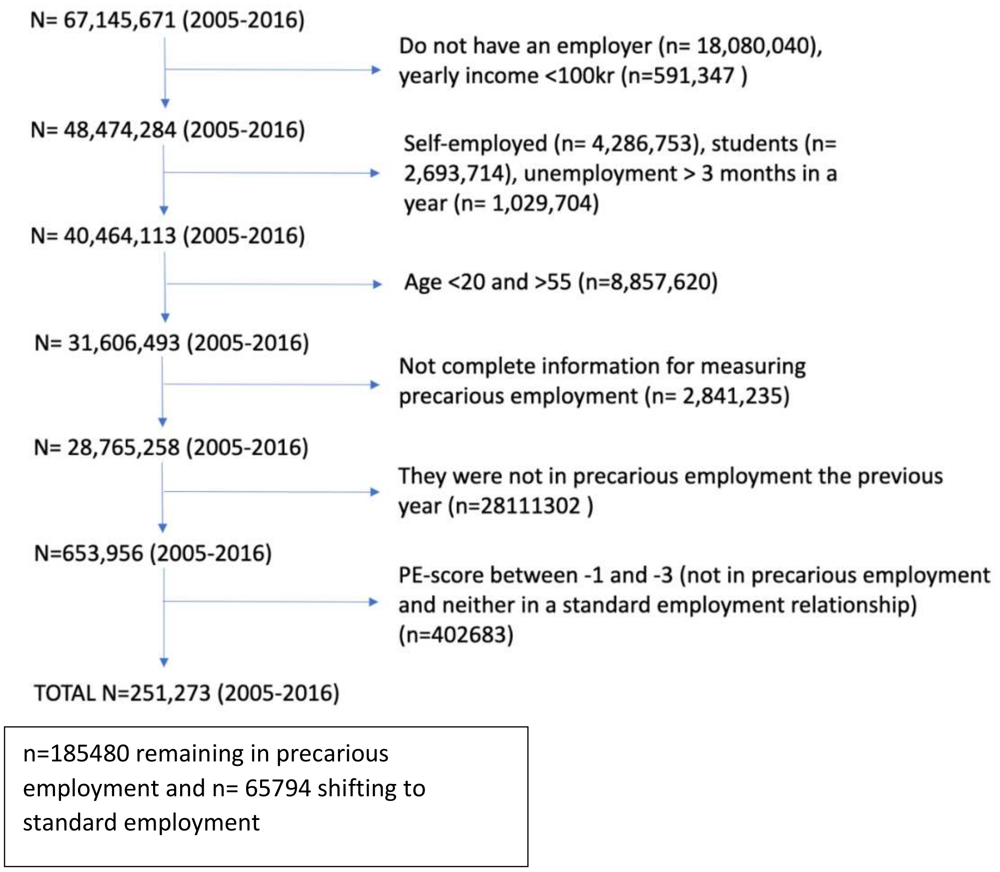

Note: The initial N refers to observations of individuals over the years. The sample for each year is of approximately 5 million people (5 million per 12 years of follow-up).

Table S1. Sample size at baseline for all the series of target trials included

	**2005**	**2006**	**2007**	**2008**	**2009**	**2010**	**2011**	**2012**	**2013**	**2014**	**2015**	**2016**	**TOTAL**
Continuation on precarious employment	12547	10437	14251	17272	17034	17657	17328	16800	16302	15737	15400	14714	185479
Shifting to standard employment	3536	2356	8472	7298	7249	6064	5118	5982	5462	4906	4714	4624	65794
TOTAL (series of 12 target trials)	16083	12803	22723	24570	24284	23721	22446	22782	21764	20644	20115	19338	251273

Matilla-Santander N, *et al. J Epidemiol Community Health* 2023; 77:736–743. doi: 10.1136/jech-2023-220734

Figure S2. Directed Acyclic Graph (DAG) for baseline confounders.

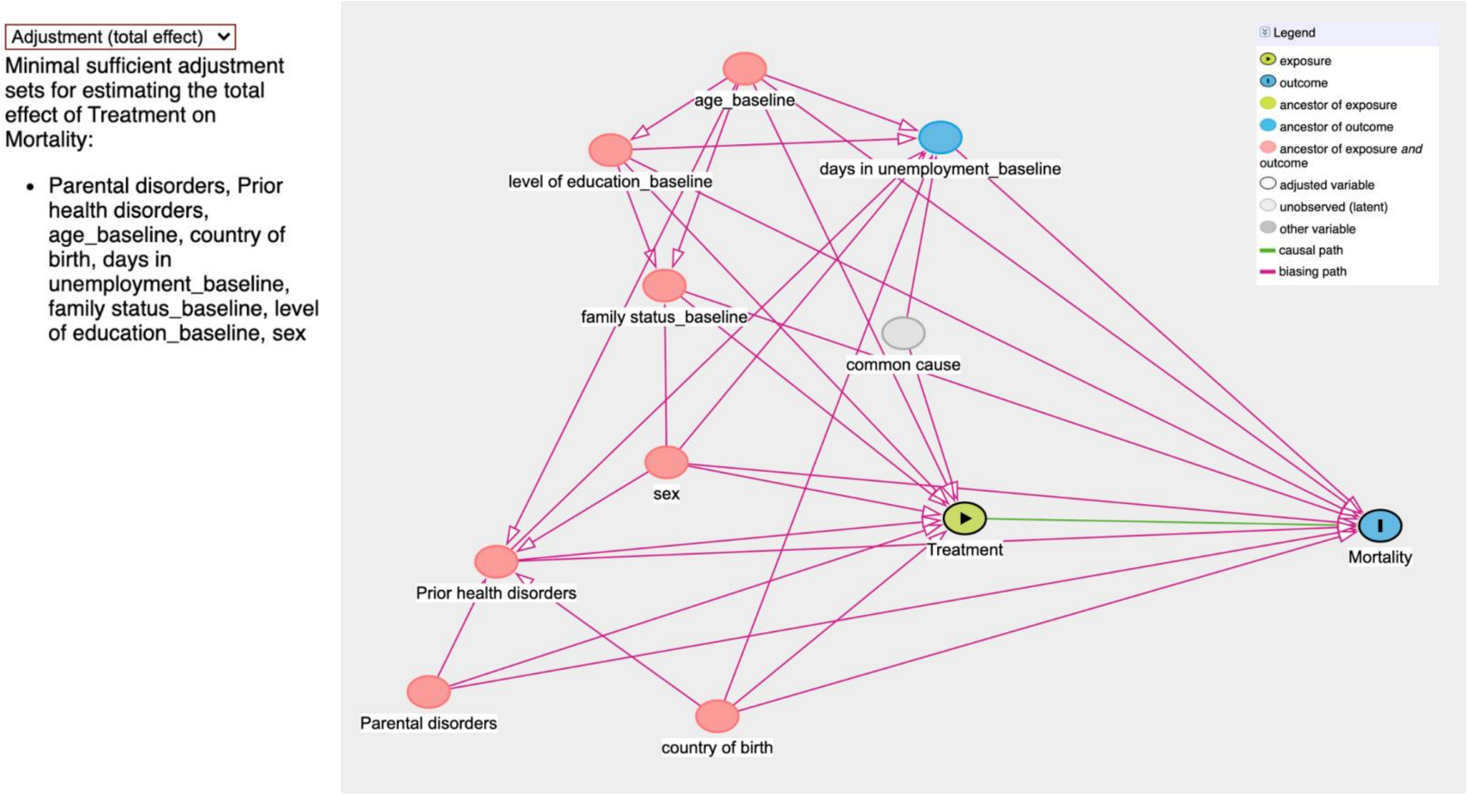

Note: Treatment is the exposure variable and corresponds to remaining in precarious employment or shifting into standard employment.

Matilla-Santander N, *et al. J Epidemiol Community Health* 2023; 77:736–743. doi: 10.1136/jech-2023-220734

Table S2. Adherence to the assigned exposure at baseline

	Adherence to the assigned exposure at baseline												
**Assigned exposure at baseline**	**Time 1**	**Time 2**	**Time 3**	**Time 4**	**Time 5**	**Time 6**	**Time 7**	**Time 8**	**Time 9**	**Time 10**	**Time 11**	**Time 12**	**TOTAL**
**Continuation on precarious employment**	185479/ 185479 (100%)	52983/17 0068 (31·1%)	29187/15 4123 (18·9%)	19443/1 21576 (14·1%)	13966/1 21576 (11·5%)	10466/1 04715 (10%)	7697/87 423 (8·8%)	5506/699 04 (7·9%)	3789/53 088 (7·1%)	2317/361 48 (6·4%)	1334/22 207 (6%)	675/120 87 (5·6%)	28.8%
**Shifting to standard employment**	65794/6 5794 (100%)	43760/ 60988 (71·7%)	33744/56 153 (60·1%)	29990/5 1141 (58·6%)	26525/4 5649 (58·1%)	23077/3 9653 (58·2%)	20196/3 4542 (58·5%)	16675/28 517 (58·5%)	12536/2 1334 (58·8%)	8145/141 19 (57·7%)	2974/57 88 (51·4%)	1670 (48·2%)	66·7%

Note: time corresponds to the year of follow-up. Time 1= baseline

Table S3. Estimated six and 12-year standardized incidence differences and risk ratios for all-cause mortality for age groups 20 to 39 years old, and 40 to 55 years old, comparing initiation to standard employment with no initiation among precariously employed workers, 2005-2017 (n= 251273).

		20 to 39 years old at baseline		40 to 55 years old at baseline	
		Remaining in precarious employment	Shifting to standard employment	Remaining in precarious employment	Shifting to standard employment
Deaths n(%)		364 (0.30%)	116 (0.24%)	880 (1.41%)	200 (1.11 %)
Intention-to-treat effect [(a)]	6-year mortality incidence per 1000 persons (95%CI)	2.49 (2.29;2.70)	2.22 (1.91;2.54)	12.8 (12.1;13.5)	10.06 (7.73;12.4)
	6-year mortality incidence difference (95%CI)	ref	-0.26 (-0.63;0.10)	ref	-2.75 (-5.13;-0.37)
	12-year mortality incidence per 1000 persons (95%CI)	7.48 (6.81;8.15)	5.71 (4.40;7.01)	33.3 (31.7;35.1)	30.8 (27.7;33.9)
	12-year incidence difference (95%CI)	ref	-1.78 (-3.27;-0.28)	ref	-2.49 (-5.83;-0.86)
	RR (95%CI)	ref	0.84 (0.68;1.04)	ref	0.83 (0.71;0.97)
Per-protocol effect [(b)]	6-year mortality incidence per 1000 persons (95%CI)	2.37 (1.59;3.15)	2.32 (1.80;2.85)	15.8 (11.6;19.9)	8.97 (3.18; 14.8)
	6-year mortality incidence difference (95%CI)	ref	-0.05 (-0.95;0.85)	ref	-6.80 (-12.9;-0.62)

[a] Comparing initiation to standard employment vs. no initiation at baseline. [b] Comparing initiation to standard employment at baseline and continuation over follow-up. The 12-year mortality incidence and incidence difference is not shown for the per-protocol effect, due to very low adherence and therefore low cases, which produced unreliable effects.

Note. RR: adjusted for age, level of education, health diagnoses, family composition, unemployment spells at baseline. Mortality incidence and differences were standardized to the joint distribution of the baseline covariates for the intention-to-treat effect. For the per-protocol effect they were also weighted for time-varying confounders.

Matilla-Santander N, *et al. J Epidemiol Community Health* 2023; 77:736–743. doi: 10.1136/jech-2023-220734
